# Rethinking the conceptual terrain of AIDS scholarship: lessons from comparing 27 years of AIDS and climate change research

**DOI:** 10.1186/1744-8603-5-12

**Published:** 2009-10-06

**Authors:** May Chazan, Michael Brklacich, Alan Whiteside

**Affiliations:** 1 Department of Geography and Environmental Studies, Carleton University, Loeb B 349, Colonel By Drive, Ottawa, Canada; 2 Health Economics and HIV/AIDS Research Division, University of KwaZulu-Natal, Durban, South Africa

## Abstract

**Background:**

While there has recently been significant medical advance in understanding and treating HIV, limitations in understanding the complex social dimensions of HIV/AIDS epidemics continue to restrict a host of prevention and development efforts from community through to international levels. These gaps are rooted as much in limited conceptual development as they are in a lack of empirical research.

**Methods:**

In this conceptual article, the authors compare and contrast the evolution of climate change and AIDS research. They demonstrate how scholarship and response in these two seemingly disparate areas share certain important similarities, such as the "globalization" of discourses and associated masking of uneven vulnerabilities, the tendency toward techno-fixes, and the polarization of debates within these fields. They also examine key divergences, noting in particular that climate change research has tended to be more forward-looking and longer-term in focus than AIDS scholarship.

**Conclusion:**

Suggesting that AIDS scholars can learn from these key parallels and divergences, the paper offers four directions for advancing AIDS research: (1) focusing more on the differentiation of risk and responsibility within and among AIDS epidemics; (2) taking (back) on board social justice approaches; (3) moving beyond polarized debates; and (4) shifting focus from reactive to forward-looking and proactive approaches.

## Background

In the 27 years since the first cases of AIDS were recorded, HIV/AIDS has become one of most highly studied diseases in history. Epidemics continue to grow, albeit unevenly, and impacts are escalating, reaching beyond individuals and families to pose major challenges to development broadly. This is most obvious in southern Africa, where antenatal prevalence levels in some countries are over 30 percent. While there has been significant medical advance in understanding and treating HIV, the complex and place-specific social, economic, cultural, behavioural and psychological dimensions remain a puzzle.

Limitations in understanding these social dimensions, which in turn restrict a range of HIV/AIDS prevention and response efforts, are rooted as much in limited conceptual development as in a lack of empirical research. HIV/AIDS scholars have tended to conceptualize "impacts" as sequential and short-term effects resulting from the virus, rather than considering the complexities and inter-generational dimensions of epidemics and their consequences [1,2].

Likewise, some AIDS researchers and advocates point to the limitations inherent in popular "techno-fix" responses (e.g. the focus on microbicides and circumcision at the 2006 International AIDS conference), noting the continued challenges involved in understanding and changing the underlying social structures that fuel the uneven spread and burden of AIDS epidemics [3,4]. This paper specfically addresses these and other key conceptual limitations through a novel comparative analysis of historical trends and contemporary debates within HIV/AIDS and climate change scholarship.

Research on AIDS and on climate change share certain similarities. Scholars in both areas are struggling to understand phenomena that are unprecedented, complex and highly dynamic, and that have different impacts on different people and places. In both, "social vulnerability" is emerging as a key scholarly theme (e.g., [5,6]). Research on these two major world issues has followed similar trajectories, starting from a physical or life sciences perspective and working to integrate social sciences. There exist conceptual overlaps, similar limitations, and the beginnings of a dialogue between development practitioners and researchers in these two fields. This is especially true in southern Africa, as evidenced by partnering of climate change and AIDS specialists in such research initiatives as Southern Africa Vulnerability Initiative (SAVI) and Regional Network on AIDS, Livelihoods and Food Security (RENEWAL).

This paper compares and contrasts the evolution of climate change and AIDS research, suggesting that scholars can learn from a comparative analysis of key debates and trends within climate change and AIDS scholarship. It addresses four conceptual limitations in the AIDS field: (1) the "globalization" of AIDS discourses and the associated masking of uneven vulnerabilities to infection and impact; (2) the highly medical framing of AIDS and tendency to seek technical solutions; (3) the polarization of debates within the field; and (4) the crisis-orientation that has characterized AIDS research and response.

The paper outlines the evolution of, and current trends in, each area of study. It explores parallels and divergences between AIDS and climate change research, noting especially the forward-looking and longer-term focus of climate change research and the sophistication of social vulnerability concepts in this field. It ends by suggesting opportunities for advancing AIDS research. (It is noteworthy that this is largely a one-way analysis looking at extending AIDS research. While a similar analysis examining the ways in which AIDS scholarship could provide insights to climate change scholars would be equally worthwhile, this is beyond the scope of this paper.)

This *conceptual *paper is suggesting that conceptual overlaps and differences within AIDS and climate change research may provide insights into future HIV/AIDS scholarship. It does not seek to study AIDS and climate change together in any substantive way, nor does it argue for links between HIV spread and climate change or vice versa.

## Discussion

### HIV/AIDS Research and Response: Over 25 Years

**Figure 1:**
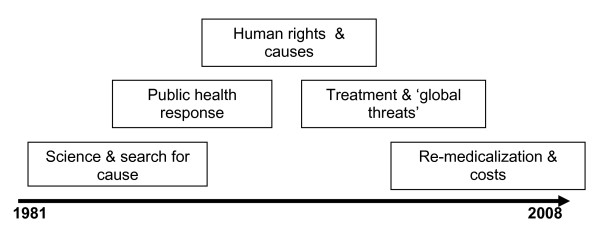
**Dominant Research Themes in HIV/AIDS**.

To understand the central limitations within contemporary AIDS scholarship and, ultimately, suggest ways in which climate change research could inform these, a basic historiography is required. The history of AIDS research and response can be divided into the early years (1981-1996) and the later years (1996-2008). This is not an all-inclusive review (for lengthier and more comprehensive accounts, see [7]); rather, based on key research papers, policy documents, international responses, and major conferences, this section aims to anchor the major concepts examined in this paper within their historical, intellectual and political foundations. The dominant research themes from 1981 to 2008 are summarized in Figure 1 [adapted from [7,8]].

#### The early years 1981 - 1996

The unusual clustering of the disease that make up AIDS was first recognised in 1981 in the USA, and growing numbers of similar immune deficiency diseases were soon identified in Europe, Australia, New Zealand and Latin America. In central Africa, health workers were observing ailments not previously seen: reports of deaths from wasting in Uganda [7]; Kaposi's sarcoma (a cancer) in Zambia [9] and cryptococcosis (an unusual fungal infection) in Kinshasa [10]. In July 1982, the disease was officially named Acquired Immune Deficiency Syndrome (AIDS), and in 1983 the cause, the human immunodeficiency virus (HIV), was identified.

Early research was dominated first by the medical/life sciences, and then by public health and epidemiology. Scientists sought to understand what was causing the disease and how it was transmitted in order to prevent its further spread, alleviate symptoms, prolong lives and, ultimately, eradicate the virus. Early responses were scientific and technical (and prevention-oriented): improving blood safety, providing condoms, encouraging safe injection practices, and searching for potential treatments and vaccines.

It quickly became apparent that medical/technical approaches were insufficient, as no cure or vaccine could be readily developed, and providing condoms did not lead to the widespread adoption of safer sexual practices. Thus, by the 1990s, AIDS research began to shift away from its initial medical, scientific and technical foundations: there was growing scholarly interest in the individual, social, and economic milieu that lead to vulnerability to HIV infection, and a recognition that social justice, poverty and equity issues were driving the uneven spread of the virus within and between communities and societies [11,12].

Among the pioneers of this shift was Dr. Jonathan Mann, head of the Global Programme on AIDS in the World Health Organisation (WHO). In 1986 he began deploying teams to developing countries, to start national AIDS programmes [13]. This was the first sign of international institutional focus on the social (and equity) dimensions of the epidemic. It is noteworthy, however, that outside of the WHO, AIDS was not yet placed on the agenda of any United Nations (UN) agencies; indeed, international responses between 1986 and 1996 were characterized by denial, underestimation, and over-simplification (i.e., conceptualizing HIV/AIDS solely as a medical issue)[14]. It was not until the end of this period that the work of Dr. Mann gained wider ascendancy, and social scientists, activists, and international advocates called heavily upon human rights approaches in understanding and responding to the epidemic (see Figure 1). Interestingly the recent WHO report on the social determinants of health reflects this thinking, which is ignored in HIV[15].

Thus the first 15 years of the epidemic may be summarised as follows:

• The first response, combining an epidemiology and public health perspective, aimed at understanding transmission, who was at risk, and how the spread could be prevented.

• Once the virus was identified, science sought treatments and biomedical answers. Alongside this were attempts to prevent the spread by promoting safer sex and injecting practices.

• By the end of the period, human rights approaches were gaining ascendancy; attention turned to *why *people are exposed to HIV. Despite growing numbers of deaths, however, there was little focus on broader social and economic impacts.

#### The later years: 1996 - 2008

By 1996, there were major changes in response to HIV/AIDS, reflecting and reflected in much of the scholarship. There was a shift from the previous "science-epidemiology" focus to a proliferation of scholarship and institutional interest around understanding the social and economic dimensions of epidemics. The new UN agency charged with co-ordinating the response to the epidemic - UNAIDS - began operations in Geneva in 1996, acknowledging the need for comprehensive responses to AIDS epidemics, and recognizing that such multi-faceted (social, economic, behavioural, developmental, medical) responses reached beyond realm of "health."

This shift away from the medical/technical focus did not last long, however. The same year, at the XI International AIDS Conference in Vancouver, it was announced that effective new drugs to treat AIDS had become available. The result was a swell of interest in medical interventions; but with costs running at $12000 per patient per year concerns around unequal and inequitable access emerged. By the XIII International AIDS Conference in Durban in 2000, these issues were squarely on the agendas of all involved in HIV/AIDS.

Responses to AIDS have since been dominated by new initiatives for making treatment accessible, especially in developing countries. This led to a re-medicalization of HIV/AIDS and increasing international pledging of resources (see Figure 1). The development of generic drugs meant the price of medicine had fallen to about $100 per patient per year by 2008. In 2001, UN Secretary General, Kofi Annan called for spending on AIDS to be increased ten-fold in developing countries, the Global Fund for AIDS, TB and Malaria was established, and President George W. Bush pledged $15 billion toward his Presidential Emergency Programme for AIDS Relief (PEPFAR). In 1996 there was about $300 million for HIV/AIDS in low and middle income countries; by 2008 this increased to $13.7 billion[16]. Among this international mobilization, concerns for social drivers and underlying vulnerabilities were largely subsumed by renewed hope for medical solutions.

With the turn of the millennium, discourses around AIDS also became increasingly "globalized" (that is the impacts of AIDS in developing countries were deemed an issue of "global concern"). The globalization of AIDS discourses and the impetus for global response were further propelled by an international trend toward securitization and a language of "global threats". In 2000, United States vice president, Al Gore said: "it (HIV) threatens not just individual citizens, but the very institutions that define and defend the character of a society. ... It strikes at the military, and subverts the forces of order and peacekeeping." The US National Intelligence Council then produced its "The Global Infectious Disease Threat and Its Implications for the United States"[17]. Six months later, the UN Security Council passed Resolution 1308, stating: "the HIV/AIDS pandemic, if unchecked, may pose a risk to stability and security"[3].

Whether based in sound evidence or not, this dialogue marked an important shift in thinking about HIV/AIDS as an epidemic that could potentially have widespread implications among even the most affluent and powerful. Most recently, with the continued pledging of large sums of money, this "globalization" in the conceptualization of AIDS impacts and responses has extended into concerns over "global governance" (to be discussed further in the section that follows).

The hallmarks of the past 12 years were:

• Treatment became available and prices of drugs plummeted. With this, scholarly concerns with social drivers and underlying vulnerabilities were largely overtaken by enthusiasm for treatment and renewed hope in medical intervention.

• The level of resources grew rapidly and new global initiatives were announced.

• The language of security and threat to global order was used, resulting in a further globalization of AIDS response and discourse.

• However, the number of infections continued to rise, especially in southern Africa.

### HIV/AIDS: Current Themes

The particular orientation of AIDS response and scholarship outlined above has in turn prompted a multidimensional and vibrant field of research and scholarly debates. Four current trends are outlined below; these will be revisited at the end of the paper in order to suggest future directions for AIDS research.

#### Theme 1: Tendency toward "globalized" discourses

As discussed above, in HIV/AIDS arenas, discourses have become "globalized". This manifests in the language of "global threats" which is still used (e.g., the 2006 AIDS, Security and Conflict Initiative (ASCI)). Issues of "global governance" have become central as large sums of money are pledged. Moreover, in AIDS research the "globalized" language is clearly captured in a shift that took place in the 1990's - the majority of international organizations shifted gear from an earlier focus on isolated "epidemics" (be they among homosexual, intravenous drug users or African populations) toward concerns for the "global AIDS ***pan***demic". This globalized discourse, however, misses the differentiated nature of both the spread of the epidemic and its impact. There remains a tension faced by those working in the field of AIDS: finding a balance between overstating the case (and potentially homogenizing what is a very uneven and differentiated 'threat') and the risk of understating it (or limiting who feels compelled to take responsibility) and risking getting fewer resources [18].

#### Theme 2: Re-medicalisation and scientisation

Also discussed above, with the development of effective treatments in the mid-1990s, the tendency towards a (re)-medicalisation of the epidemic gained momentum. This was further propelled by the decline in drug prices and the increased flow of resources. Besides the initial focus on antiretroviral treatment (ARV), the re-medicalization of AIDS research and response saw renewed hope placed on other potential medical and technological "solutions", such as vaccines, microbicides and circumcision. Although there has been continual expectation of breakthroughs, there are still no solutions. Resources keep on being poured into scientific/medical research through new avenues such as the International AIDS Vaccine Initiative and by the new philanthropies such as the Gates Foundation marking an overwhelming international desire for a scientific or technological "fix" to HIV/AIDS.

#### Theme 3: The polarization of debates

Throughout the history of AIDS research and response, there has been a tendency toward polarized debates, depicted by a series of "either-or" framing of responses (i.e. debating the need for treatment *versus *prevention, as has happened among multilateral institutions, or the need for drugs *versus *nutrition, as has been the case in many African contexts, most notably in South Africa [19]). In addition, among an international contingent of "dissident" scientists and politicians, there has been a questioning of both the data collected on HIV/AIDS and the science itself [20].

#### Theme 4: Focus on crisis intervention

The evolving response of the AIDS field has focussed on intervening in developing crises. As new clusters of diseases emerged, the emphasis was on understanding the epidemiology and biology. As the syndrome began to spread, prevention and behaviour came into focus. As issues of equity and discrimination emerged, human rights were placed on the agenda. As treatment became available, discussion around costing, access and patenting grew. Most recently, as the magnitude of illness and death in parts of Africa is beginning to have society-wide effects, attention is turning toward understanding impacts. The overarching trend is that AIDS research been reactive, historically - it has focused predominantly on crisis intervention, not on understanding the complex and place-specific drivers of infection and impacts. Notable exceptions include Campbell and Stillwaggon [21,22].

Despite this tendency, some scholars have identified AIDS as a "long-wave event" recognizing that AIDS epidemics can take over 100 years to work through society[23]. Thus, impacts from the current epidemic will last decades. The notion that AIDS is a long-wave event and the conceptual implications of this for social vulnerability research will be discussed in subsequent sections.

### An Evolution of Climate Change Research

Climate change research has also matured considerably over the past 27 years, although until very recently there have been limited interactions between climate change and HIV/AIDS communities. This section provides an overview of key concepts that have framed climate change research. The existence of anthropogenic climate change is now well established (for example, see [24,25]) thus we do not attempt to summarize this research here. Instead, we purposefully review the evolution of four major themes within climate change research.

The creation of an international agency to address climate change occurred about eight years, prior to the creation of UNAIDS. With growing evidence that human activities were altering the Earth's climate throughout the 1980s, the Intergovernmental Panel on Climate Change (IPCC) was created in 1988 by the World Meteorological Organization (WMO) and the United Nations Environment Program (UNEP) with the mandate to assess scientific, technical and socio-economic information. IPCC is employed in this paper as a window into climatic change research; its four assessments, in 1990, 1995, 2001 and 2007, provide an effective mirror of research trends in this field [24,26-30]. Note that although IPCC is highly influential in both research and policy (e.g., has been co-awarded the Nobel Peace Prize), and although it employs a meticulous peer review process (David Suzuki Foundation[31]), its assessments remain controversial among some scientists [32,33]. Nevertheless, given the comprehensiveness of the IPCC assessment process (one for which there is no parallel within AIDS research), we have selected to draw heavily on this in our review of climatic change research; this is not intended as a commentary on IPCC as an institution or on its major findings.

**Figure 2:**
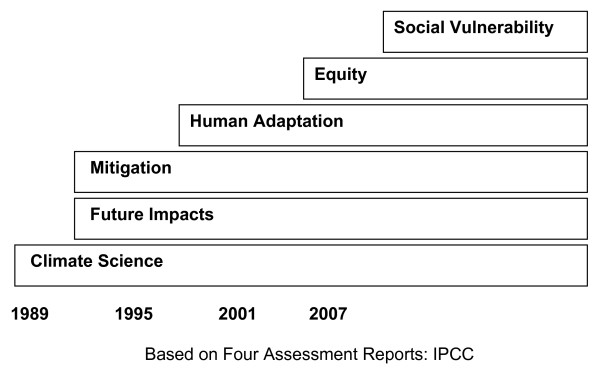
**Trends in Climatic Change Research**. 1989, 1995, 2001, 2007.

Figure 2[34,35] summarizes the progression of key themes within the four IPCC reports. As this schematic indicates, the IPCC has always adopted a forward-looking approach; understanding the potential impacts of *future *climate change has been a central theme since 1989.

Figure 2 also indicates that, as with the HIV/AIDS field, the IPCC's foundations are based in science, in this case physical climatic sciences including the reconstruction of past climates, the understanding of current climates, and the projection of future climates. The first two reports were dominated by climate sciences, reflected in the rapid evolution of broad-scale modelling throughout the 1980s and 1990s.

Although social science contributions lagged behind the development of the physical sciences, there has been a proliferation of this research recently. In the first two IPCC reports, the social sciences focused on climate *mitigation *options - looking at how to reduce greenhouse gas (GHG) emissions or recapture and sequester carbon generated by human activities[36]. By the mid-1990s, research into climate change *impacts *(i.e., how climate change is affecting and will affect different communities) and *human adaptation *(i.e., how people are able to respond to various stresses in their environments) was underway. The uncertainties associated with future impacts on a wide range of economic activities were featured in the 1989 report, while the technical feasibility of both mitigation (reducing/limiting greenhouse gases (GHG)); and adaptation (finding ways to reduce potential impacts through technical means or by changing where and how certain communities live), only began to emerge by 1995.

Since the mid-1990s, multiple calls to refocus social science contributions have sparked considerable research, especially in the areas of equity [37,38] and social vulnerability [5,39]. Equity issues have emerged in at least two related ways: first, attention to achieving more fair representation within the science communities participating in climate change research (so that research coming out of non-Western institutions are given voice in international assessments); second, increasing awareness about the dislocation between countries which are contributing to climate change and countries that will be adversely impacted [40-42].

These trends within the IPCC has directly contributed to the growing awareness that those most responsible for causing climate change are not the ones who are most likely to bear the greatest negative consequences. Indeed, a key finding from the Small Island States chapter in 2001 was as follows: "The small island states account for less than 1% of global GHG emissions but are among the most vulnerable of locations to the potential adverse impacts of climatic change and sea-level rise" [41].

With growing concerns around the inequitable distribution of potential impacts, came the need to better understand what makes certain groups and places particularly vulnerable and determines how effectively they respond to potential stresses. While the underlying (and uneven) social, economic, political and geographical factors driving what has become known as "social vulnerability" were virtually absent from the first two IPCC assessments, this has now emerged as a central issue in the field. This is reflected in a number of ways: the recent chapter title, *Climate Change Impacts, Adaptation and Vulnerability *[29]; each regional chapter in the third assessment included a vulnerability subsection; and the third report concluded with a chapter on *Vulnerability to Climate Change and Reasons for Concern *[43].

Vulnerability is defined in the third assessment as "the degree to which a system is susceptible to, or unable to cope with, adverse effects of climatic change" [44] In the most recent report, social vulnerability concepts are expanded - vulnerability is understood to be exacerbated by the presence of other stresses (such as entrenched poverty and weak governance) and to be affected by the extent to which future development efforts are equity-oriented, sustainable and culturally-sensitive [45]. The most recent report concluded that poorer communities (some of which are more dependent on climate-sensitive resources such as local water and food supplies) tend to have limited adaptive capacities, and therefore are disproportionately vulnerable [29].

Although contemporary climate change science has not abandoned its physical science roots, it has clearly begun to recognize that a full understanding of climate change requires careful consideration of the interaction of human and climate systems. Nevertheless, even with growing attention to equity and social vulnerability in the research arena, the trend in climate change response continues to lean toward technological solutions. The section in the most recent report entitled *Mitigation of Climate Change *[30] focused on the application of existing technologies - potential "techno-fixes" like switching from coal-fired power to renewable energy sources, improving energy efficiency in buildings, and introducing more effective economic incentives to support mitigation efforts. Full-out reduction in consuming fossil fuels, particularly among affluent communities, remains rather silenced in current discussions.

Moreover, the "globalness" of climate change - the global-scale nature of the science and the discourses of global-scale threats - was a major factor leading to IPCC's creation in 1989 and remained a primary concern in 2007. This is evident in the most recent report, which assesses the extent to which "impacts may change at larger increases in global mean temperature," focusing on *worldwide *impacts that may occur as *average *temperatures rise [46]. By focusing on "the global", however, the chapter follows much of the popular and academic climate change discourse: it obscures the regional variability that is expected to characterize future changes in climate as well as the unevenness in response capacities across and within nations. Climate change is a global phenomenon, but the preoccupation with this perspective diverts attention away from the unevenness in GHG emissions and the social and political inequities that undermine the response capacities of the most vulnerable communities and regions.

In summary, the research is based on science; and climate change research has always been forward-looking. Early social science contributions focused on mitigating (preventing) climate change itself, while understanding how communities might adapt to change took longer to come onto the agenda (at times with some polarization between these positions); in both cases, recourse to "techno-fix" solutions continues to dominate. Several new issues have emerged over the past decade with the most notable being:

• Understanding the *uneven *capacities of human systems to adapt to climate change, and recognizing North-South *equity *issues; and

• Focusing on understanding and addressing underlying social *vulnerabilities *that put some individuals and communities in "harm's way".

### Climate Change: Current Themes

Some commonalities and differences between climate change and HIV/AIDS scholarship clearly begin to emerge from the above discussion. Before turning specifically to an examination of these, this section highlights four current themes within the climate change field. As in the discussion around HIV/AIDS, we will revisit these themes in the analysis and conclusion sections of this paper.

#### Theme 1: Science and uncertainty

The uncertainty inherent in understanding climate change has important research, response and political implications. Given the complexities involved, it is unreasonable to expect firm predictions of future climates and climate-society relationships. It is in this context that climate change research is gradually placing more emphasis on living with uncertainty. For example, there has been a proliferation of "scenario" exercises designed to articulate future uncertainties about how human activities will alter GHG emissions and climate regimes [47]. Among a small but growing group of researchers, there has also been emphasis on understanding vulnerabilities to environmental changes broadly, and on intervening to reduce these vulnerabilities now, regardless of debates around future climate scenarios [48].

The uncertainty around climate change has also been used towards political ends - deploying it as a delaying or diversionary tactic to deter response efforts. This "denialism", not unlike the AIDS denialism discussed earlier (i.e., recourse to data debates and pseudo-scientific "evidence" that HIV does not cause AIDS), includes well-constructed arguments denying human activities are contributing climate change, as well as calls for more science to determine the extent to which the climate change reflects natural or human-induced variability. Denialism invariably commences with reference to the Earth's climate as dynamic (i.e., that it has alternated between warm and cool periods for over 500,000 years) and suggests that climate science is in need of further development [32,33,49]. While denialism is steadily losing ground, it does continue to exist and is often employed to protect large emitters of GHGs.

#### Theme 2: "Global" versus "local" and equity perspectives

The "globalness" of climate change lies at the heart of the issue: even more so than HIV/AIDS, climate change is overwhelmingly framed as a "global threat." The prevailing belief that no one will escape its consequences has indeed prompted many affluent leaders to take action. Climate change is clearly a global issue requiring global solutions. Much like in the AIDS field, however, the continued globalized discourses around climate change stand in contrast to, and can even serve to mask, the uneven and inequitable vulnerabilities that are emerging as key concerns.

#### Theme 3: Leaning toward technical interventions

Much of the social science-based research has focused on either reducing GHG emissions or sequestering atmospheric carbon in order to reduce the magnitude of future climate changes. Technical interventions such as carbon trading schemes, incentives to encourage a more efficient use of fossil fuels, and switching to non-carbon fuels have been thoroughly researched and are routinely included as key components in climate change programs.

Attempts to alter human behaviours which underly the problem (e.g., urban North Americans driving large vehicles and other overly consumptive indulgences), or to enhance communities' capacities to adapt to climate change (e.g., by finding strategies to make them less dependent on fragile, resource-dependent economies), have received less attention and are more controversial from a public policy perspective. As discussed in the HIV/AIDS field, technical solutions continue to dominate; however, attempts to bridge what has been a polarization between mitigation (ie technical solutions) and adaption (ie social solutions) is giving way to a more balanced approach which does not pit mitigation against adaptation.

#### Theme 4: Focus on the future

As discussed above, climate change research has traditionally been framed in a forward-looking context (and here we are beginning to see a departure from the crisis-orientation that has characterized HIV/AIDS research and response). For example, paleoclimatology investigates past climates, but it is routinely framed as basic research into Earth system processes which provides a window into future climates. In addition, the use of scenarios to depict a range of futures has been and continues to be standard practice in climate change research and recognizes human activities are bound to change to many stimuli.

Moreover, more recent social vulnerability research attempts to understand what present-day conditions cause certain people to be hardest hit by environmental changes and to be the least able to respond to these stresses. This too is forward-looking, in that it aims to find ways to prevent future climate change impacts by reducing present-day vulnerabilties.

## Conclusion

### Summary and Ways Forward: Parallels, Divergences and Directions

The above discussion reveals a number of the parallels and divergences in how researchers and practitioners have responded to and conceptualized HIV/AIDS and climate change. In this final section, we will explicitly summarize these key parallels and divergences, and then extend these points so as to suggest future conceptual directions for AIDS research.

#### Conceptual parallels: summarizing three key trends

Three parallel conceptual trends in HIV/AIDS and climate change research are pivotal for understanding current conceptual limitations in the AIDS field. The first is the tendency toward globalized discourses in both areas, which have often masked the differentiated risks and responsibilities associated with both HIV/AIDS epidemics and human-induced climate change. In the AIDS field, the shift to globalized language took place in the late 1990s, coinciding with the ascendancy of "global threats" and security discourses. While this "globalization" of AIDS discourses served to mobilize international actors, it also functioned to minimize massive inequalities in vulnerabilities within and between countries and communities. As Marais notes with respect to AIDS, *"*In this fanciful world, we're somehow all bobbing in 'the same boat', if not exactly equally than all equally-at-peril..." [1] The reality, however, is that AIDS is not really a 'global' problem - at least uniformly so. He illustrates stark inequalities in who is most vulnerable and who will be most impacted within the South African context, and he notes that these inequalities increase manyfold when considering HIV/AIDS at an international level.

In the climate change arena, we have seen the tendency to frame the 'threats' in a similarly homogenizing way. Reference to the Earth as a unified system reinforces similar images of "all bobbing in the same boat." [50]. As in the AIDS arena, such a globalized discourse does assist in mobilizing international audiences, however, negative consequences of climate change are not, and will not be, even, and the problem, again, is that the heavy focus on the "globalness" can mask these very uneven vulnerabilities.

The second important parallel is that both AIDS and climate change research have developed from scientific perspectives: AIDS from life sciences, virology and epidemiology; and climate change from Earth and paleo-climatic sciences. In both cases, this leaning and continued emphasis (or reinvigoration, as in the case of AIDS) has resulted in tendencies to de-politicize the issues and focus on scientific or technical solutions. Just as focussing on vaccines, circumcision, and microbicides does not delve into the very difficult task of social change that will undoubtedly be required to curb the spread of HIV and mitigate the impacts of AIDS, carbon trading, alternative energy schemes, and projects to fortify dams are all part of a comprehensive response to climate change but still overlook the key question: who is consuming and who will pay the price? In both the climate change and AIDS arenas, there is a tendency to avoid tackling vested interests (such as removing power from oil lobbies) and difficult issues (such as gender inequalities and sexual violence).

The third parallel trend is the issue of "denialism". "Data debates" and "denialism" have taken place in both arenas: around AIDS, debates (especially in southern African contexts where epidemics are most widespread) have often focussed on scrutinizing prevalence levels, questioning the causes of AIDS, and being skeptical of the utility of ARVs; in the climate change arena, there has been ongoing debate as to the amount of change attributable to "natural" and "human-induced" causes, as well as skepticism that fossil fuel burning is at the core of the pending problem. It is difficult to understand why this "denialism" has taken place, though perhaps the unprecendented and uncertain nature of both phenomena, alongside not knowing what to do and attempting to protect the status quo, is partially to blame. The result has been a polarization within the debates in both fields, which distracts from the underlying issues and the associated inequities, and thus hinders mobilization for change that will improve the circumstances of those most vulnerable.

To summarize, three key parallel trends within AIDS and climate change research include: (1) the tendency toward globalized discourses and the masking of uneven vulnerabilities; (2) the dominance of scientific perspectives and continued grasping for "techno-fixes"; and (3) the polarized debates and resulting diversion away from equitable and comprehensive responses. Together, examining these parallels begin to elucidate why the task of understanding the *root causes *of the *uneven *impacts of AIDS (and of climate change) has not been (or has failed to remain) at the forefront of dominant research or development agendas. This analysis begins to shed light on one of the central conceptual limitations addressed in this paper and thus highlights ways in which scholars and practitioners in the AIDS field might begin to reorient their approaches - prioritizing the underlying, place- and time-specific drivers of uneven vulnerabilties.

#### An extended look at one divergence: toward a forward-looking vulnerability approach in AIDS research

There are also some obvious differences in the evolution of the AIDS and climate change fields. The key divergence discussed in this paper offers an important way AIDS scholars could learn from the work of their climate change colleagues. This major difference can be summarized as follows: while climate change research has always been forward-looking, attempting to predict and prevent future impacts, AIDS research has tended to be crisis-oriented, focusing on understanding impacts as they happened.

AIDS research and response has followed the evolution of the disease and the epidemic. The key issues changed as the epidemic spread and treatment became available. This approach is retroactive - researchers, health care professionals and policy makers look backward to understand, and in some cases intervene in, AIDS impacts. Climate change research, by contrast, has focused on reducing future impacts. This has meant not only modeling predicted climate changes, but within a smaller cluster of the most recent social science research, moving beyond this to understanding present-day vulnerabilities in order to help vulnerable groups respond to future stresses [51,52].

This forward-looking vulnerability approach is at the crux of the conceptual reorientation advocated in this paper. Growing in popularity among social scientists in the climate change field, the approach places emphasis on understanding the specific social, economic, political and geographical factors that currently make some people and groups vulnerable to any new shock or stress in their environment, including, but not limited to those caused by present-day and future climate change. It recognizes these vulnerabilities as exisiting now, before the worst of predicted climate change has hit; as Kelly and Adger [48] explain, "the vulnerability of any individual or social grouping ... is determined primarily by their existent state... rather than by what may or may not happen in the future". These existing vulnerabilities therefore afford places to intervene, regardless of knowing with certainty how much sea level will rise or where storms will surge. This approach strives to be preventative, bypassing polarized debates over future impacts - it also speaks precisely to the limitation outlined above, seeking to understand drivers of uneven vulnerabilities. A lesson for AIDS researchers thus emerges: vulnerabilities to HIV infection and AIDS impacts exist *now*, before AIDS epidemics have fully run their course; thus, understanding these existing context-specific vulnerabilities gives opportunities to intervene in *proactive *ways.

**Figure 3:**
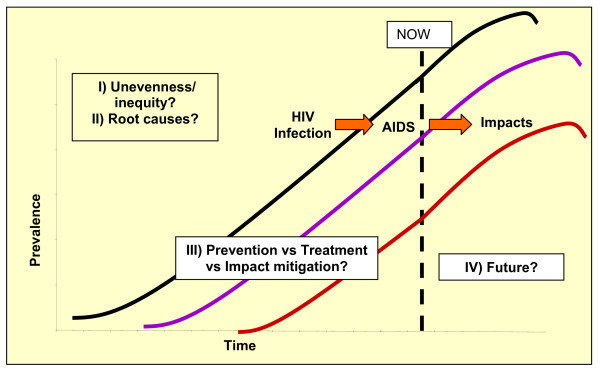
**Emerging Challenges for AIDS Research**. Prevalence, Time.

Some, but only very few, AIDS scholars have stressed this need for proactive and preventive measures to understand and mitigate potential future impacts [53,54]. As indicated earlier, the important temporal dimension of HIV/AIDS has been highlighted by scholars describing the "long-wave" nature of epidemics (See Figure 3). The three curves depicted in Figure 3[3] indicate that in any generalized AIDS epidemic (such as those spreading through southern and eastern Africa), time lags can be expected between the points at which: (a) infection levels begin to climb, (b) there is an epidemic of people exhibiting symptoms of full-blown AIDS, and (c) society-wide impacts such as orphaning can be measured. This is because HIV takes several years to progress from causing asymptomatic infection, to manifesting in acute illness, to killing its host. Thus, this conceptualization recognizes the need to look forward: given the high levels of HIV infection in parts of the world, AIDS, like climate change, will have effects well into the future.

However, the projection depicted in Figure 3 does not capture the four key points highlighted on this illustration and emphasized throughout this paper: (1) the inequities inherent in AIDS impacts (this projection does not show differentiation in impact within or between societies); (2) the root causes of vulnerabilities to any of the three curves; (3) where or how to intervene (are efforts best placed on preventing HIV spread, lengthening times between infection and illness through treatment, attempting to mitigate impacts, or some combination of these, often polarized, strategies); or (4) what actions will alter future trajectories (they should not be considered fixed or unchangeable). Thus, while the projection reveals the start of an important shift toward forward-looking AIDS research, taken alongside certain insights from the climate change arena, it also represents and reinforces emerging and continued challenges in the AIDS field.

#### Conclusion: key challenges for AIDS research

This paper was premised upon the notion that examining certain key parallels and divergences within and among AIDS and climate change research and response could offer new insights for AIDS scholarship. The overarching question must now be addressed: what can we really learn from how scholars have grappled with climate change and AIDS, and what does this mean for AIDS scholarship?

The four key themes highlighted above, and examined throughout the discussion, are central to answering this question:

1. We have seen parallels in the globalized framing of both HIV/AIDS and climate change. Yet, it is clear that AIDS epidemics (as well as climate change impacts) are uneven and differentiated. Nuance is needed: one approach could therefore be to frame HIV/AIDS epidemics in terms of their unevenness (probing the questions of who is most vulnerable, who is most affected, who is most able to respond, and why), while at the same time recognizing that it is in part *because of this unevenness *(not because of some "real" or otherwise phantom security threat) that a global response is appropriate.

2. We have also seen a re-medicalization in the AIDS field, with a focus on treatment, resources and techno-fixes. This discussion hence suggests that AIDS scholars take back on board social justice approaches, which were more prominent a decade ago but have since been pushed to the margins of the field. This does not mean moving away from treatment, as treatment is also a social justice issue (particularly where access is concerned), and certainly medical interventions will need to be part of any comprehensive response. Rather, drawing on the work of colleagues in the climate change arena, this analysis suggests looking at *what causes inequalities *in infection levels, capacity for response, impacts *and *access to treatment.

3. In both areas we have seen polarization and data debates. Clearly in the AIDS field there is a need to move beyond treatment versus prevention and other such "either-or" debates. What underlying issues cut across unequal access to treatment, risk of infection *and *likelihood of bearing impacts? Can we intervene in underlying drivers common in all of these areas? Perhaps some of these crosscutting root causes are gender inequalities, social marginalization or livelihood insecurity? How are such root causes unique to specific places or similar across different communities? These complex questions pose a major challenge to social scientists in the field, but are crucial to consider in order to devise and implement effective and comprehensive responses.

4. Finally, one central lesson AIDS scholars can take from the climate change literature is a reorientation in the way they think about vulnerability and impact. As in recent social vulnerability research, it would make sense to push for an AIDS agenda that looks forward - an agenda that seeks to understand present-day vulnerabilities in order to reduce future impacts, in a *preventive *rather than reactive way. We note AIDS epidemics have not yet run their course, and thus impacts will inevitably continue to unfold to decades; the magnitude and distribution of these future impacts depend, however, on existing vulnerabilities; and although major social and structural changes will almost certainly be required, these vulnerabilities can be reduced now in order to reduce or prevent hardships.

By examining the conceptual similarities and differences within AIDS and climate change research, this paper has provided challenges toward an emerging AIDS research agenda. Many of the challenges facing the AIDS field are not dissimilar to those scholars are grappling with elsewhere. Indeed, there are opportunities to learn from climate change research, as we have demonstrated. There is a need to move beyond what often is constructed as "issues-based" silos to examine barriers within social inquiry more broadly.

## Abbreviations

AIDS: Acquired Immune Deficiency Syndrome; ASCI: AIDS, Security and Conflict Initiative; ARV: Antiretroviral Treatment; GHG: Greenhouse gas; HIV: Human Imunodeficiency Virus; IPCC: Intergovernmental Panel on Climate Change; PEPFAR: Presidential Emergency Programme for AIDS Relief; RENEWAL: Regional Network on AIDS, Livelihoods and Food Security; SAVI: Southern Africa Vulnerability Initiative; UN: United Nations; UNAIDS: Joint United Nations Programme on HIV/AIDS; UNEP: United Nations Environment Program; WHO: World Health Organisation; WMO: World Meteorological Organization.

## Competing interests

The authors declare that they have no competing interests.

## Authors' contributions

MC conceived of this paper and engaged MB and AW in dialogue around their respective fields, climate change and HIV/AIDS. MC outlined the study in a presentation format; MB and AW contributed feedback and substantive intellectual input; and MC presented this preliminary version at the 2006 International AIDS Conference in Toronto, Canada. MC then drafted the manuscript; AW contributed to sections pertaining to HIV/AIDS; MB contributed to climatic change sections. All authors edited and proofread the final manuscript.
